# Association Between Systemic Inflammatory Response Index at Labour Admission and Episiotomy Wound Dehiscence After Vaginal Delivery

**DOI:** 10.3390/diagnostics16193198

**Published:** 2026-10-01

**Authors:** Zeynep Güvenç, Mehmet Efe Namlı, Hande Kurt Güven, Elif Yılmaz

**Affiliations:** 1 Department of Obstetrics and Gynecology, Keşan State Hospital, Edirne 22800, Türkiye; 2 Department of Obstetrics and Gynecology, Ankara Ataturk Sanatoryum Training and Research Hospital, Ankara 06010, Türkiye

**Keywords:** episiotomy, wound dehiscence, perineal trauma, systemic inflammatory response index, discrimination, predictive value of tests

## Abstract

**Background/Objectives:** We examined the association between the systemic inflammatory response index (SIRI) measured on admission in labour and complete episiotomy wound dehiscence, and whether the SIRI adds useful discrimination beyond available covariates. **Methods:** This retrospective case–control study included 200 cases and 200 controls attending postpartum clinics at a tertiary maternity unit (September 2019–September 2022). Controls were randomly sampled from eligible attendees on the same clinic days. The SIRI was calculated from pre-delivery blood counts. Unconditional logistic regression adjusted for maternal age, primiparity, education, income, birthweight and gestational age. Important intrapartum and repair variables were unavailable. **Results:** The median SIRI was 3.00 in cases and 2.52 in controls (*p* < 0.001). The log-transformed SIRI was associated with dehiscence after adjustment (OR 1.49 per doubling, 95% CI 1.13–1.97; *p* = 0.005), whereas the untransformed term was not statistically significant (*p* = 0.063). The SIRI had poor discrimination (AUC 0.601, 95% CI 0.544–0.655). Adding the SIRI increased the reference-model AUC from 0.699 to 0.713 (ΔAUC 0.014, 95% CI 0.001–0.039); adding neutrophil count yielded an AUC of 0.712. **Conclusions:** In this selected clinic-attending population, the SIRI showed a model-dependent association with dehiscence but insufficient discrimination for individual risk prediction. Residual confounding remains possible. The small incremental gain does not support using the SIRI for clinical risk stratification or treatment decisions.

## 1. Introduction

Episiotomy practice varies between settings, and studies of clinician attitudes and selective versus routine use illustrate the influence of local practice on its frequency [1,2]. Wound infection and dehiscence are important complications after the procedure. Identifying women who may require closer postpartum assessment is clinically relevant, but any proposed marker must provide information beyond routinely available obstetric characteristics.

Reported dehiscence rates are difficult to compare because definitions and follow-up methods differ. A systematic review of 15 studies found risks ranging from 0% to 22%; studies with clinical assessment at one to three weeks postpartum reported 9.3%, compared with 3.6% in studies without such assessment [3]. These estimates include second-degree tears as well as episiotomies and do not specifically describe complete episiotomy dehiscence. Perineal wound complications can impair recovery and may require further treatment [4]. The REPAIR trial reported fewer clinically relevant wound complications with prophylactic oral antibiotics [5]. This supports research into prevention, but does not establish how a blood-based marker should guide treatment.

The SIRI is calculated as the neutrophil count multiplied by the monocyte count and divided by the lymphocyte count. It can be derived from a routine complete blood count without additional sampling. The SIRI has been investigated in early pregnancy loss [6,7], gestational diabetes [8,9], intrahepatic cholestasis [10], neonatal outcomes [11] and pregnancies complicated by antiphospholipid syndrome [12]. Such studies provide a rationale for examining the SIRI in other obstetric settings; they do not establish its value for predicting perineal wound healing. In particular, a between-group association may coexist with substantial overlap in individual values. The relevant questions are whether the SIRI discriminates between outcomes and whether it adds information to available clinical variables or to its component blood-cell counts.

Accordingly, this study addressed three related questions. First, is the SIRI measured on admission in labour associated with subsequent complete episiotomy wound dehiscence after adjustment for available clinical and sociodemographic covariates? Second, how well does the SIRI discriminate between women with and without dehiscence? Third, does calculating the SIRI provide useful additional discrimination compared with the available covariates or the neutrophil count alone?

## 2. Materials and Methods

### 2.1. Design, Setting and Participants

This was a single-centre, retrospective case–control study conducted in the Department of Obstetrics and Gynecology of Dr. Sami Ulus Maternity and Children’s Health Training and Research Hospital, a tertiary referral maternity unit in Ankara, Türkiye. Reporting follows the STROBE recommendations for observational studies [13], and the analysis of predictive performance was informed by TRIPOD+AI [14] and by current guidance on the evaluation of clinical prediction models [15].

The study sampling frame comprised women who gave birth vaginally with a mediolateral episiotomy between 1 September 2019 and 1 September 2022 and attended the same unit’s postpartum clinic. Both cases and controls were drawn from this clinic-attending population. Local practice was to discharge uncomplicated women 24 h after birth and arrange reviews at one and three weeks postpartum. At review, women underwent history taking, perineal inspection, vaginal examination and pelvic ultrasound. Women who did not attend follow-up were not represented; the sampling frame should therefore be distinguished from all women delivering at the centre. The common follow-up setting supports comparability of clinical assessment, but does not ensure that attendees represent the broader population of women undergoing episiotomy.

Cases were women with documented complete episiotomy dehiscence during postpartum follow-up. Complete dehiscence was defined as separation involving the skin, underlying muscle and vaginal mucosa; superficial or partial separation was not counted. Controls had documented intact healing and no recorded dehiscence through their final available postpartum assessment. Wound assessment was performed by the clinicians providing care in the unit’s postpartum clinic. These clinicians were unaware of the calculated SIRI values at assessment. Blinding to the component complete blood count results was not documented. The same anatomical definition was used for retrospective case classification, but the available records did not permit verification of uniform examination procedures across clinicians or throughout the study period. No standardised scoring system, such as REEDA (redness, oedema, ecchymosis, discharge and approximation), was used, and assessor-level information and interobserver agreement were unavailable.

The same exclusions applied to both groups: no episiotomy; caesarean birth; chronic systemic disease, including diabetes mellitus, hypertension or connective tissue disease; current or past smoking; body mass index above 30 kg/m^2^; immunosuppressive disease or therapy; continuous medication use; absence of postpartum review data; or absence of a complete blood count before delivery. These restrictions resulted in a selected population with low comorbidity. Applicability to women with these common risk factors for inflammation or impaired wound healing is limited.

#### Study Size and Control Selection

No formal a priori sample-size calculation was performed. The study included the 200 consecutive eligible cases identified in the available postpartum records during the study period and 200 controls, giving a 1:1 ratio. Precision was assessed using 95% confidence intervals around association and discrimination estimates. The number of identified cases should not be interpreted as a complete count of all dehiscence events among women who delivered at the centre.

Controls were randomly sampled from eligible women attending the same postpartum clinic on the same clinic days as the cases. This was intended to limit temporal differences in clinical practice and follow-up and to draw controls from the setting in which cases were identified. Individual case–control pair identifiers were not retained in the original dataset. Matched sets and clinic-day strata could not be reconstructed from the available study material. Consequently, a conditional analysis of matched sets could not be performed. We used unconditional logistic regression with adjustment for the available covariates and describe the analysis as an unmatched case–control comparison. This approach does not reproduce a matched analysis or remove possible residual confounding by calendar time.

The numbers of women screened and excluded at each stage, the total number of deliveries, the number of vaginal deliveries with episiotomy and the number attending postpartum follow-up during the study period were unavailable in the study records. Neither a centre-specific episiotomy rate nor a complete-dehiscence incidence could therefore be estimated. A valid incidence estimate would require a defined population of women with episiotomy and sufficiently complete ascertainment of subsequent dehiscence; an all-delivery denominator alone would not resolve this problem. The absence of these counts also prevents assessment of the representativeness of included women. Multivariable adjustment addresses only recorded covariates, and bootstrap correction addresses model optimism.

### 2.2. Data and Laboratory Methods

Data were extracted from the hospital electronic record. Venous blood was obtained on admission in labour, before delivery, collected into EDTA tubes and analysed on a Sysmex XN-1000 haematology analyser (Sysmex Corporation, Kobe, Japan). The interval from labour onset to sampling, labour stage or cervical dilatation at sampling, timing relative to membrane rupture and interval from sampling to delivery were not available for analysis. Thus, sampling was defined by admission in labour rather than a standardised physiological stage. These were intrapartum measurements obtained before delivery, not baseline inflammatory measurements obtained before labour. From the neutrophil, monocyte, lymphocyte and platelet counts we derived SIRI = neutrophils × monocytes ÷ lymphocytes; neutrophil-to-lymphocyte ratio (NLR); monocyte-to-lymphocyte ratio (MLR); platelet-to-lymphocyte ratio (PLR); systemic immune-inflammation index (SII) = platelets × neutrophils ÷ lymphocytes; and pan-immune-inflammation value (PIV) = neutrophils × platelets × monocytes ÷ lymphocytes. All component counts were expressed as ×10^9^/L. The interval from episiotomy to documented dehiscence was recorded in whole days. SIRI was the primary inflammatory exposure; neutrophil count and the other indices were secondary comparators.

Maternal and partner education were categorised as university, high school, or primary/secondary. Household income was recorded relative to the national minimum wage (above, at, or below). One control had a gestational age recorded as 3 weeks; this implausible value was set to missing without imputation, leaving 399 women in models containing gestational age. The other variables reported in Table 1 and Table 2 were complete. Instrumental vaginal delivery, duration of the second stage, duration of ruptured membranes, number of vaginal examinations, operator experience, repair technique and suture material were not available in the analytic dataset. Instrumental delivery was therefore unrecorded, not confirmed to be absent. The adjusted models could therefore account only for the recorded covariates, leaving possible confounding by unmeasured obstetric and repair-related factors.

### 2.3. Statistical Analysis

Continuous variables were inspected graphically and assessed for distributional shape. Because the haematological variables were right-skewed, they are presented as median (interquartile range) and compared using the Mann–Whitney U test; rank-biserial correlation was used to quantify between-group effect size. Categorical variables were compared using Pearson’s χ^2^ test.

SIRI was the prespecified primary inflammatory exposure. Its right-skewed distribution motivated natural-log transformation, and odds ratios were expressed per doubling by rescaling the coefficient by ln(2). This specification assumes a constant change in log odds for each doubling of SIRI; skewness alone does not establish that the log-linear form is correct. Unconditional multivariable logistic regression included maternal age, primiparity, maternal education, household income, birthweight and gestational age. These variables were selected a priori on clinical grounds. Their combination is termed the multivariable reference model because it does not include several established intrapartum predictors and is not a comprehensive clinical prediction model.

Sensitivity analyses used SIRI as an untransformed continuous term and in quartiles, with the lowest quartile as the reference. These specifications assess sensitivity to the exposure scale but do not establish the shape of the underlying relationship.

Discrimination was quantified using the area under the receiver operating characteristic curve (AUC), with 95% confidence intervals estimated from 2000 bootstrap resamples. The discriminatory performance of SIRI was compared directly with that of the neutrophil count, its simplest major cellular component. Other derived inflammatory indices were evaluated as secondary comparators. Paired bootstrap resampling was used to estimate differences in AUC between markers.

Incremental discrimination was evaluated by comparing the multivariable reference model, the reference model plus log-transformed SIRI, and the reference model plus neutrophil count. Differences in AUC were estimated using paired bootstrap resampling, and optimism was assessed using bootstrap internal validation. Improvement in model fit was assessed with a likelihood-ratio test. Neither improved fit nor an AUC increment was treated as evidence of clinical net benefit, and similar AUC estimates were not interpreted as demonstrating equivalence. These comparisons did not establish clinically validated risk groups or management thresholds.

The 1:1 case–control sampling fixed the proportion of cases at 50%, so sample-derived probabilities, calibration and predictive values cannot be interpreted as estimates for the source population without appropriate correction. Calibration and decision-curve analysis can, in principle, be evaluated using methods that account for case–control sampling, but require suitable information on target-population prevalence or sampling fractions. Such information was unavailable; population calibration and clinical net benefit were therefore not evaluated. For illustration, positive and negative predictive values (PPV and NPV) were calculated with Bayes’ theorem at hypothetical prevalences while holding the observed sensitivity and specificity fixed. These projections do not provide external validation or quantify uncertainty in sensitivity, specificity or transportability. The Youden-derived SIRI threshold of 3.54 and the alternative threshold of 2.58 were treated as exploratory; neither was evaluated in an independent sample. Two-sided *p* values < 0.05 were considered statistically significant. Results for secondary indices and thresholds were interpreted as exploratory. All analyses were performed using Python version 3.12 (Python Software Foundation, Wilmington, DE, USA), with statsmodels version 0.14.6, scikit-learn version 1.8.0 and SciPy version 1.17.1.

### 2.4. Ethics

The study was approved as a residency thesis by the Clinical Research Ethics Committee of Ankara Atatürk Sanatoryum Training and Research Hospital on 23 August 2023 (approval 2012-KAEK-15/2778; institutional reference E-53610172-799-224084845). Because the design was retrospective and used routinely collected, de-identified records, the committee waived the requirement for individual informed consent. The study conformed to the Declaration of Helsinki.

## 3. Results

### 3.1. Participants

Four hundred women were included: 200 with complete episiotomy dehiscence and 200 with intact healing (Table 1). The underlying numbers of deliveries, episiotomies, postpartum attendees, screened records and exclusions were unavailable. Consequently, a complete participant flow diagram and centre-specific dehiscence incidence could not be provided. The 50% case proportion reflects the sampling design, not the frequency of dehiscence at the centre. All 400 women contributed to the descriptive comparisons; 399 contributed to models containing gestational age after one implausible value was set to missing (Section 2.2).

Women with dehiscence were younger (median 25 vs. 27 years, *p* < 0.001) and more often primiparous (72.0% vs. 50.5%, *p* < 0.001). The groups also differed in maternal education and household income: university education was recorded in 24.0% of cases and 38.5% of controls (*p* = 0.003), and income above minimum wage in 49.0% and 66.5%, respectively (*p* = 0.002). There was no statistically significant between-group difference in gestational age or birthweight.

Dehiscence was recognised a median of 6 days after birth (mean 6.77 ± 1.95, range 2–13). It presented within the first five days in 31 women (15.5%), between days 6 and 10 in 156 (78.0%), and after day 10 in 13 (6.5%).

### 3.2. Haematological Parameters

Neutrophil counts, white cell counts, SIRI, NLR, MLR, SII and PIV were all higher in the dehiscence group; lymphocytes, monocytes, platelets and haemoglobin were not (Table 2). The differences were small. The SIRI, the index with the largest separation, had a median of 3.00 in cases against 2.52 in controls: a ratio of 1.19, and a rank-biserial correlation of 0.201. The corresponding figures for the other indices were smaller still.

Despite the statistically significant between-group difference, the SIRI distributions overlapped substantially. Thirty-eight per cent of controls had SIRI values above the case median, and 36% of cases had values below the control median. The association therefore corresponded to limited separation of individual women.

Median white blood cell counts were 10.4 × 10^9^/L in cases and 10.0 × 10^9^/L in controls, within the reported pregnancy reference interval of 5.7–14.4 × 10^9^/L from six weeks onward [16]. Only 5.3% of the sample exceeded that upper limit. The cited study excluded blood tests obtained during the week before labour; its interval therefore provides context rather than a validated intrapartum reference limit. These values should be interpreted in the context of pregnancy and labour rather than labelled pathological leukocytosis on the basis of non-pregnant reference ranges.

### 3.3. Discrimination

All indices showed limited discrimination (Table 3; Figure 1). The SIRI had an AUC of 0.601 (95% CI 0.544–0.655), corresponding to approximately 60% correct ranking of a randomly selected case–control pair. The threshold of 3.54, selected by the Youden index in this sample, gave 42.0% sensitivity and 76.0% specificity (LR+ 1.75; LR− 0.76). At the alternative threshold of 2.58, sensitivity was 63.0% and specificity 54.0% (LR+ 1.37; LR− 0.69). The PIV and neutrophil count had AUCs of 0.596 and 0.587, respectively. The PLR confidence interval included 0.5 (AUC 0.526, 95% CI 0.471–0.585). Optimism correction changed the SIRI AUC estimate by 0.001. These threshold-specific estimates describe performance in the development sample and do not establish clinical cutoffs.

### 3.4. Adjusted Association and Incremental Discrimination

Under the log-linear specification, each doubling of the SIRI was associated with higher adjusted odds of dehiscence (OR 1.49, 95% CI 1.13–1.97; *p* = 0.005; Table 4). Relative to the lowest quartile (SIRI ≤ 2.08), adjusted ORs were 1.71 (95% CI 0.94–3.13), 1.91 (1.05–3.47) and 2.36 (1.29–4.33) for quartiles 2–4 (*p* for trend = 0.006). The untransformed linear term gave an adjusted OR of 1.10 per unit (95% CI 0.99–1.22; *p* = 0.063). The point estimates were in the same direction, but statistical significance depended on the functional form. A per-unit OR and a per-doubling OR represent different exposure contrasts and should not be compared as if they measured the same increment. These results do not establish a nonlinear relationship or confirm the log-linear form as correct.

Primiparity was associated with dehiscence (adjusted OR 2.39, 95% CI 1.43–3.97; *p* < 0.001). Relative to income above minimum wage, adjusted ORs were 1.92 (95% CI 1.11–3.32; *p* = 0.020) for income at minimum wage and 3.32 (95% CI 1.01–10.89; *p* = 0.048) for income below minimum wage. The lowest income category contained only 23 women (13 cases and 10 controls), and its estimate was imprecise. These income associations are exploratory. The unadjusted association with maternal age (OR 0.91 per year) was attenuated after adjustment (OR 0.99, 95% CI 0.92–1.05); this does not identify which covariates account for the attenuation.

The multivariable reference model had an AUC of 0.699. Adding the log-transformed SIRI increased the AUC to 0.713, an absolute increment of 0.014 (95% CI 0.001–0.039), and improved model fit (likelihood-ratio χ^2^(1) = 8.09; *p* = 0.005). Optimism-corrected AUCs were 0.672 and 0.685, respectively. Adding neutrophil count instead yielded an apparent AUC of 0.712 (Figure 1). The near-identical point estimates do not establish equivalence or non-inferiority. Although the confidence interval for the AUC increment after adding the SIRI excluded zero, the small increment does not establish a clinically useful improvement in individual risk prediction. Population calibration and clinical net benefit were not evaluated.

### 3.5. Hypothetical Predictive-Value Scenarios

Table 5 and Figure 2 illustrate predictive values at assumed prevalences for the exploratory threshold of 2.58, using sensitivity of 63.0% and specificity of 54.0%. At an assumed prevalence of 9.3%, 1000 women would include approximately 59 true-positive, 417 false-positive, 34 false-negative and 490 true-negative results. The projected PPV is 12.3% and NPV 93.4%. The 9.3% scenario is informed by studies with clinical postpartum assessment [3]; it is not an estimate for our centre or specifically for complete episiotomy dehiscence. At the same assumed prevalence, the threshold of 3.54 would yield a projected PPV of 15.2%, while missing 58% of cases. These calculations illustrate the consequences of the observed sensitivity and specificity under fixed assumptions. They are not externally validated estimates of predictive accuracy, and actual performance may differ with patient spectrum, follow-up and outcome definition.

## 4. Discussion

### 4.1. Principal Findings and Clinical Interpretation

In this retrospective case–control sample, a higher SIRI was associated with complete episiotomy wound dehiscence after adjustment under the log-linear specification. The association was sensitive to how the SIRI was modelled, and residual confounding by unrecorded intrapartum and repair factors cannot be excluded. The SIRI alone had poor discrimination (AUC 0.601), while its addition to the reference model increased the AUC by only 0.014. The present data do not support using the SIRI for individual clinical risk stratification.

The comparison with neutrophil count is relevant because the SIRI combines three routinely measured cell counts. Its standalone AUC was 0.601, compared with 0.587 for neutrophils, and models augmented with the SIRI or neutrophil count had similar apparent AUCs (0.713 and 0.712). This pattern suggests that the composite may contribute little beyond its neutrophil component. However, numerical similarity is not proof of equivalent performance, and these data do not identify the biological source of the association. The SIRI does not require an additional blood sample or assay when a differential blood count is already available, but it requires combining three measurements rather than reading a single reported count. That extra calculation would be clinically worthwhile if it improved risk estimates or management decisions; neither benefit was demonstrated here. These findings also do not support substituting neutrophil count as a validated risk marker, because its standalone discrimination was poor.

The overlap in SIRI values explains why a statistically significant between-group difference offers limited individual discrimination. Both explored thresholds had modest likelihood ratios. At an assumed prevalence of 9.3%, the lower threshold yielded a projected PPV of 12.3%, with substantially more false-positive than true-positive results. The Youden threshold improved specificity but reduced sensitivity to 42.0%. Neither threshold was validated independently. Their use should remain descriptive, and the hypothetical predictive values should not be presented as performance expected in a particular maternity unit.

### 4.2. Limitations and Applicability

Residual confounding is a central limitation of the adjusted SIRI association. Instrumental delivery, duration of the second stage of labour, duration of membrane rupture, number of vaginal examinations, operator experience, repair technique and suture material were unavailable. These factors may influence tissue injury, bacterial exposure or wound healing independently of the measured inflammatory cell profile. If they differed between women with higher and lower SIRIs, they could account for part of the observed association; the direction and magnitude of this influence cannot be determined. The adjusted OR therefore describes an association conditional on the recorded covariates and does not establish that the SIRI has an independent biological effect on healing.

Primiparity was associated with dehiscence in our adjusted model, but direct comparison with published estimates requires attention to the variables included and the outcome definition. A recent meta-analysis identified assisted vaginal birth as a prognostic factor for perineal wound complications (OR 2.77, 95% CI 1.89–4.06) [4]. In a retrospective study of 84 cases and 249 controls, Puissegur and colleagues found that instrumental delivery and a prolonged second stage remained associated with early wound breakdown after adjustment [17]. These findings make their omission from our dataset consequential. Adjustment for age, parity, socioeconomic variables, birthweight and gestational age cannot substitute for direct measurement of intrapartum trauma and repair-related factors.

Selection through postpartum attendance also limits interpretation. Both cases and controls were assessed in the same follow-up setting, which supports comparability but does not ensure representativeness. Women who did not attend follow-up were not included. Attendance may depend on wound symptoms, access to care and socioeconomic circumstances; selection on these factors could affect both the distribution of SIRI and its association with dehiscence. The findings should therefore be applied cautiously to the wider population of women undergoing episiotomy, including those who do not return for routine review.

The total delivery count, episiotomy denominator, follow-up attendance and screening exclusions were unavailable. We therefore cannot determine whether complete dehiscence was relatively frequent or infrequent at this centre, estimate incidence, or assess selection at each stage. Same-day control sampling may reduce temporal differences, but unmatched analysis cannot recover the original pairing or account for unmeasured clinic-day differences.

The income findings require separate caution. The lowest income category included 13 cases and 10 controls, and its wide confidence interval indicates considerable uncertainty. Postpartum attendance may also differ by income and wound symptoms. If women with intact wounds and greater resources are more likely to attend routine follow-up, the control group could overrepresent higher-income women. Information on non-attendees was unavailable, so this possibility could not be evaluated. We cannot infer a causal effect of income or distinguish it from unmeasured social and clinical factors. The result is hypothesis-generating and requires confirmation in a prospectively followed population.

Blood sampling introduces a further limitation. Counts were obtained on admission in labour, so the SIRI may reflect physiological leukocyte changes associated with labour as well as variation between women [16]. Labour stage or cervical dilatation, membrane-rupture status and duration at sampling, and the interval from sampling to delivery were unavailable. We could therefore neither standardise the SIRI to a common stage of labour nor examine its association within comparable sampling intervals. These unmeasured differences may contribute to variability in the SIRI and may confound the observed association if they also relate to wound complications. A single admission measurement cannot separate a pre-existing inflammatory state from changes arising during labour.

Clinicians were unaware of the calculated SIRI values, but blinding to routine haematology results was not documented. The absence of standardised wound scoring and assessor-level reliability data leaves the possibility of misclassification. Excluding women with obesity, smoking history and chronic disease further limits applicability to routine obstetric populations.

The association also requires caution because the untransformed and log-transformed SIRI models gave different significance results. Quartile estimates support a broadly increasing pattern but do not validate a particular functional form. The adjusted association should therefore be interpreted within the specified model rather than presented as robust to all reasonable specifications.

Finally, model fit, discrimination, calibration and clinical usefulness address different questions. The improvement in fit after adding the SIRI did not establish better risk estimates or treatment decisions. Other biomarker studies have also shown that associations can change after adjustment for routine clinical measures [18], reinforcing the need to evaluate incremental information in a relevant clinical model. Our reference model omitted important obstetric predictors, and neither external validation nor population calibration or decision-curve analysis was available. Case–control sampling does not make these evaluations impossible in principle, but appropriate prevalence or sampling information is needed to estimate absolute risk and population net benefit. Bootstrap correction does not address these gaps. The present findings cannot establish whether SIRI-guided management is better or worse than treating all or no women. That question would require a defined intervention, clinically justified risk thresholds and validation in the intended population [14,15].

## 5. Conclusions

The SIRI measured on admission in labour was associated with complete episiotomy wound dehiscence under the adjusted log-linear specification, but discrimination was poor and the incremental improvement beyond available covariates was small. A model augmented with neutrophil count had a similar AUC, and a clinical advantage for the SIRI was not demonstrated. Residual confounding, variation in sampling during labour and selection through postpartum attendance limit interpretation and generalisability. These findings do not support the current use of the SIRI to stratify individual risk, guide treatment or determine the intensity of postpartum surveillance. Prospective studies should enumerate the source population, standardise blood sampling and wound assessment, incorporate established obstetric and repair-related factors, and assess calibration, clinical utility and external validity.

## Figures and Tables

**Figure 1:**
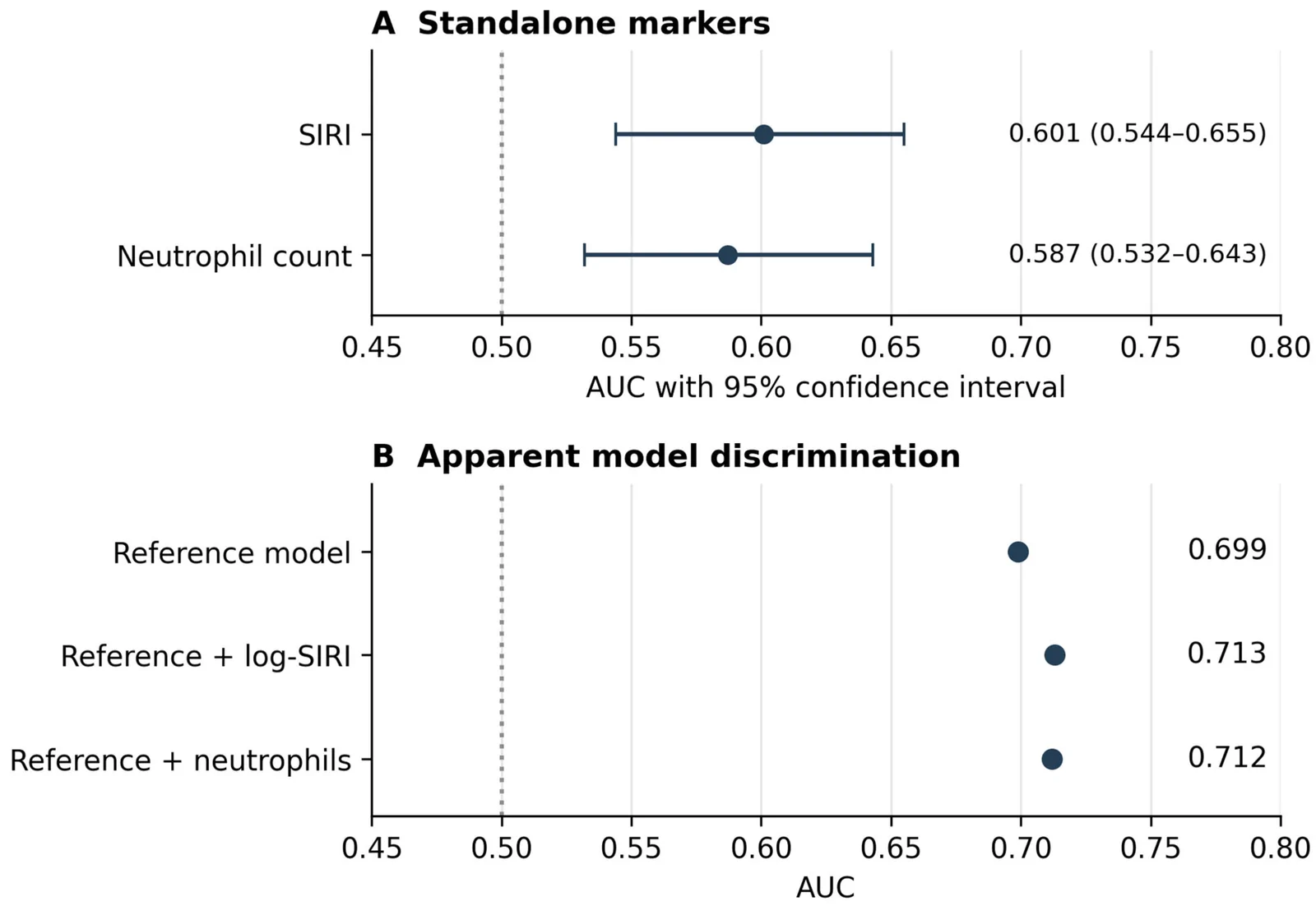
Discrimination of SIRI, neutrophil count and the multivariable models. Panel (**A**) shows the reported standalone AUC estimates with 95% bootstrap confidence intervals. Panel (**B**) shows apparent AUCs for the reference model and models augmented with log-SIRI or neutrophil count (*n* = 399). The reference model contains maternal age, primiparity, education, household income, birthweight and gestational age.

**Figure 2:**
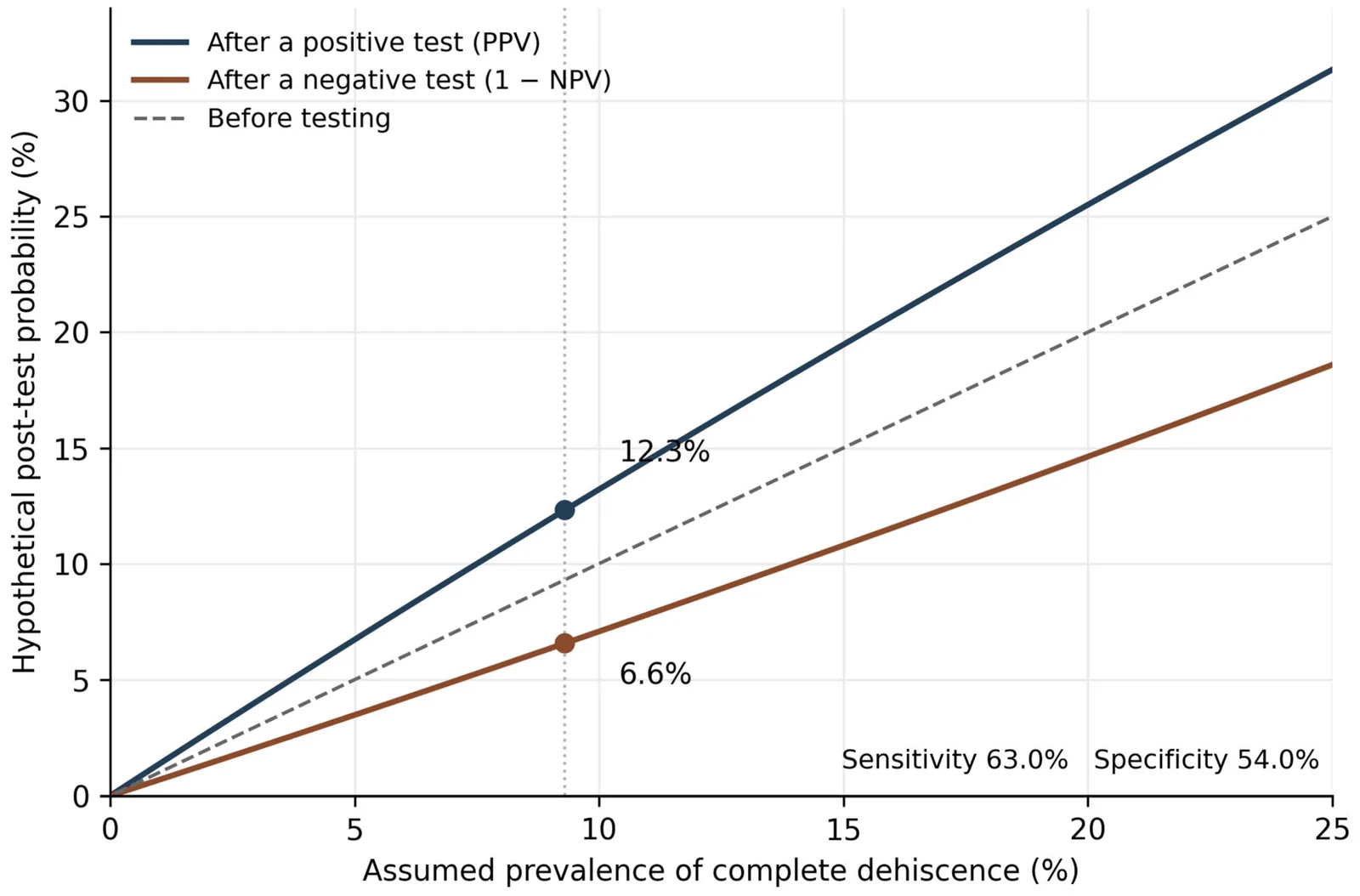
Hypothetical post-test probabilities across assumed prevalences for the exploratory SIRI threshold of 2.58. Curves are calculated with Bayes’ theorem, holding sensitivity at 63.0% and specificity at 54.0%. The positive-test curve is PPV and the negative-test curve is 1 − NPV. The diagonal denotes the assumed pre-test probability. The marked 9.3% scenario is informed by published clinical follow-up studies [3] and does not estimate the incidence of complete episiotomy dehiscence at our centre. Curves are illustrative, are not externally validated, and do not incorporate uncertainty in sensitivity or specificity.

**Table 1:** Characteristics of women with and without episiotomy wound dehiscence.

Characteristic	Dehiscence (*n* = 200)	Control (*n* = 200)	*p*
Maternal age (years)	25.6 ± 4.3/25 (23–28)	27.4 ± 4.8/27 (24–30)	<0.001
Gestational age at birth (weeks)	37.7 ± 1.8/38 (36–39)	37.7 ± 1.6/38 (36–39)	0.732
Birthweight (g)	3222 ± 352/3220 (2948–3442)	3272 ± 385/3280 (3028–3502)	0.200
Parity (number of births)	1.36 ± 0.67/1 (1–2)	1.68 ± 0.81/1 (1–2)	<0.001
Parity group			<0.001
Primiparous	144 (72.0)	101 (50.5)	
Multiparous	56 (28.0)	99 (49.5)	
Maternal education			0.003
University	48 (24.0)	77 (38.5)	
High school	131 (65.5)	98 (49.0)	
Primary/secondary	21 (10.5)	25 (12.5)	
Partner education			0.094
University	99 (49.5)	120 (60.0)	
High school	92 (46.0)	71 (35.5)	
Primary/secondary	9 (4.5)	9 (4.5)	
Household income			0.002
Above minimum wage	98 (49.0)	133 (66.5)	
At minimum wage	89 (44.5)	57 (28.5)	
Below minimum wage	13 (6.5)	10 (5.0)	
Employment status			0.110
Employed	93 (46.5)	110 (55.0)	
Not employed	107 (53.5)	90 (45.0)	

Continuous variables are shown as mean ± SD/median (interquartile range) and compared with the Mann–Whitney U test; categorical variables as *n* (%) and compared with Pearson’s χ^2^. Income is expressed relative to the Turkish national minimum wage. One control had an implausible gestational age (recorded as 3 weeks) that was set to missing.

**Table 2:** Blood count parameters and derived inflammatory indices measured on admission in labour.

Parameter	Dehiscence (*n* = 200)	Control (*n* = 200)	*p*	Rank-Biserial r
White blood cells (×10^9^/L)	10.39 (9.30–12.30)	10.02 (9.02–11.30)	0.021	0.134
Neutrophils (×10^9^/L)	7.96 (6.63–9.64)	7.26 (6.07–8.99)	0.003	0.175
Lymphocytes (×10^9^/L)	1.92 (1.52–2.35)	1.93 (1.61–2.31)	0.718	0.021
Monocytes (×10^9^/L)	0.71 (0.59–0.91)	0.67 (0.58–0.85)	0.073	0.104
Platelets (×10^9^/L)	245.00 (198.00–286.00)	236.00 (189.75–265.00)	0.204	0.073
Haemoglobin (g/dL)	11.95 (11.20–12.70)	12.30 (11.30–12.83)	0.122	0.089
SIRI	3.00 (2.25–4.21)	2.52 (1.89–3.49)	<0.001	0.201
NLR	4.05 (3.27–5.27)	3.79 (2.90–4.86)	0.020	0.135
MLR	0.38 (0.30–0.48)	0.36 (0.29–0.44)	0.024	0.131
PLR	121.60 (93.75–152.71)	120.13 (92.36–152.05)	0.362	0.053
SII	988.97 (713.63–1319.38)	871.13 (615.78–1189.48)	0.015	0.14
PIV	735.34 (470.10–1065.47)	554.75 (399.40–928.17)	<0.001	0.191

Values are median (interquartile range); *p* values are from the Mann–Whitney U test. Rank-biserial r quantifies between-group separation; values close to zero indicate substantial overlap. SIRI = neutrophils × monocytes ÷ lymphocytes; NLR = neutrophils ÷ lymphocytes; MLR = monocytes ÷ lymphocytes; PLR = platelets ÷ lymphocytes; SII = platelets × neutrophils ÷ lymphocytes; PIV = neutrophils × platelets × monocytes ÷ lymphocytes. Component counts are expressed as ×10^9^/L. Results for indices other than SIRI are exploratory.

**Table 3:** Discriminative performance of each index for episiotomy wound dehiscence.

Index	AUC (95% CI)	Exploratory Youden Threshold	Sensitivity %	Specificity %	LR+	LR−	Youden Index
SIRI	0.601 (0.544–0.655)	3.54	42.0	76.0	1.75	0.76	0.18
PIV	0.596 (0.540–0.652)	613.1	60.5	57.0	1.41	0.69	0.175
Neutrophils (×10^9^/L)	0.587 (0.532–0.643)	7.77	56.5	58.5	1.36	0.74	0.15
SII	0.570 (0.514–0.625)	988.54	50.5	61.5	1.31	0.8	0.12
NLR	0.567 (0.513–0.623)	5.1	29.5	81.0	1.55	0.87	0.105
MLR	0.565 (0.509–0.621)	0.44	38.0	74.5	1.49	0.83	0.125
White blood cells (×10^9^/L)	0.567 (0.510–0.622)	10.75	48.0	66.5	1.43	0.78	0.145
PLR	0.526 (0.471–0.585)	79.5	92.0	17.5	1.12	0.46	0.095

AUC = area under the receiver operating characteristic curve; 95% CIs were obtained from 2000 bootstrap resamples. Thresholds maximise the Youden index in this sample and are exploratory, with possible optimism from selection and evaluation in the same data. LR+ = positive likelihood ratio; LR− = negative likelihood ratio. At the alternative SIRI threshold of 2.58, sensitivity was 63.0%, specificity 54.0%, LR+ 1.37 and LR− 0.69. Neither threshold has been externally validated, and neither is supported for assigning individual clinical risk or guiding treatment.

**Table 4:** Logistic regression models for episiotomy wound dehiscence (*n* = 399).

Variable	Unadjusted OR (95% CI)	Unadjusted *p*	Adjusted OR (95% CI)	Adjusted *p*
SIRI (per doubling)	1.59 (1.22–2.07)	<0.001	1.49 (1.13–1.97)	0.005
Maternal age (per year)	0.91 (0.87–0.96)	<0.001	0.99 (0.92–1.05)	0.683
Primiparity (vs. multiparity)	2.50 (1.65–3.78)	<0.001	2.39 (1.43–3.97)	<0.001
Education: high school (vs. university)	2.17 (1.39–3.38)	<0.001	1.61 (0.93–2.79)	0.091
Education: primary/secondary (vs. university)	1.35 (0.68–2.67)	0.392	0.57 (0.22–1.51)	0.260
Income: at minimum wage (vs. above)	2.16 (1.41–3.30)	<0.001	1.92 (1.11–3.32)	0.020
Income: below minimum wage (vs. above)	1.76 (0.74–4.19)	0.198	3.32 (1.01–10.89)	0.048
Birthweight (per 100 g)	0.96 (0.91–1.02)	0.183	0.95 (0.88–1.03)	0.197
Gestational age (per week)	1.02 (0.91–1.14)	0.761	1.19 (1.00–1.41)	0.047

SIRI was modelled on the natural-log scale; its OR is expressed per doubling. The adjusted model contains all listed variables simultaneously. Reference categories are multiparity, university education and income above minimum wage. Apparent AUCs were 0.699 for the reference model, 0.713 after adding log-SIRI and 0.712 after adding neutrophil count. Optimism-corrected AUCs were 0.672 and 0.685 for the first two models. Adding log-SIRI gave ΔAUC 0.014 (95% CI 0.001–0.039) and likelihood-ratio χ^2^(1) = 8.09 (*p* = 0.005). The AUC increment does not establish a clinically useful gain in individual prediction or clinical net benefit. Similar AUCs do not establish equivalence between models. Compared with SIRI Q1 (≤2.08), adjusted ORs were 1.71 (0.94–3.13), 1.91 (1.05–3.47) and 2.36 (1.29–4.33) for Q2–Q4 (*p* for trend = 0.006). Untransformed SIRI gave an adjusted OR of 1.10 per unit (0.99–1.22; *p* = 0.063); inference is sensitive to functional form. The below-minimum-wage category contained 23 women and its association is exploratory.

**Table 5:** Hypothetical predictive values for the exploratory SIRI threshold of 2.58 at assumed prevalences.

Assumed Prevalence (%)	Scenario Interpretation	PPV (%)	NPV (%)	PPV Minus Assumed Prevalence (pp)
1.0	Illustrative assumption	1.4	99.3	0.4
3.0	Illustrative assumption	4.1	97.9	1.1
5.0	Illustrative assumption	6.7	96.5	1.7
9.3	Published clinical follow-up estimate [3]	12.3	93.4	3.0
15.0	Illustrative assumption	19.5	89.2	4.5
22.0	Illustrative assumption	27.9	83.8	5.9
50.0	Imposed case fraction only	57.8	59.3	7.8

Calculated with Bayes’ theorem while holding sensitivity at 63.0% and specificity at 54.0%. PPV = positive predictive value; NPV = negative predictive value; pp = percentage points. The final column is PPV minus assumed prevalence. The 9.3% scenario is informed by studies with clinical assessment at one to three weeks postpartum [3], but those studies included broader injury and dehiscence definitions. The other population scenarios are illustrative assumptions, not centre-specific estimates. The 50% row represents the imposed case fraction only. These projections are not externally validated and do not propagate uncertainty in sensitivity or specificity.

## Data Availability

The de-identified dataset and the analysis code are available from the corresponding author on reasonable request.

## References

[1] Yang J., Bai H. (2021). Knowledge, attitude and experience of episiotomy practice among obstetricians and midwives: A cross-sectional study from China. BMJ Open.

[2] Gu S., Pei J., Zhou C., Zhao X., Wan S., Zhang J., Adanlawo A., Luo Z., Wu G., Hua X. (2022). Selective versus routine use of episiotomy for vaginal births in Shanghai hospitals, China: A comparison of policies. BMC Pregnancy Childbirth.

[3] Perslev K., Klarskov N., Bergholt T., Jangö H. (2026). Varying incidence and definitions of infection and wound dehiscence after second-degree tear or episiotomy: A systematic review. Int. Urogynecol. J..

[4] Man R., Bhatia T., Sitch A., Morris R.K., Hodgetts Morton V., CHAPTER group (2026). Prognostic factors for wound complications after childbirth-related perineal trauma: A systematic review and meta-analysis. Acta Obstet. Gynecol. Scand..

[5] Perslev K., Klarskov N., Bergholt T., Jangö H. (2025). Risk of infection and wound dehiscence after use of prophylactic antibiotics in episiotomy or second degree tear (REPAIR study): Single centre, double blind, placebo controlled randomised trial. BMJ.

[6] Agaoglu Z., Tanacan A., Ipek G., Peker A., Ozturk Agaoglu M., Sahin D. (2024). Systemic inflammation response index and systemic immune-inflammation index for prediction of pregnancy loss: A case–control study. Reprod. Sci..

[7] Çallıoğlu N., Gül D.K., Arslan İ.Ö., Geyikoğlu İ., Demirçivi E. (2024). Inflammatory markers in systemic immune-inflammatory index and inflammatory response index to predict early pregnancy loss. Saudi Med. J..

[8] Yildiz E.G., Tanacan A., Okutucu G., Bastemur A.G., Ipek G., Sahin D. (2024). Can System Inflammation Response Index or Systemic Immune Inflammation Index predict gestational diabetes mellitus in the first trimester?. Int. J. Gynaecol. Obstet..

[9] Zhang D., Zeng Y., Sun B., Li W., Liu W., Gao H., Zhu Y., Li H., Chen Q. (2024). Inflammatory indices—Systemic immune-inflammation index (SII) and systemic inflammatory response index (SIRI)—During pregnancy and associations with gestational diabetes mellitus. J. Inflamm. Res..

[10] İpek G., Tanaçan A., Peker A., Ağaoğlu Z., Kara Ö., Şahin D. (2024). Systemic inflammation response index as a diagnostic and prognostic predictor of intrahepatic cholestasis of pregnancy: A case–control study from a tertiary center. Int. J. Gynaecol. Obstet..

[11] Kırat S. (2025). The effect of systemic inflammation on newborns: The prognostic value of the aggregate systemic inflammation index (AISI) and systemic inflammatory response index (SIRI). Diagnostics.

[12] Doğru Ş., Ezveci H., Karanfil Yaman F., Metin Ü.S., Acar A. (2025). Role of the systemic immune-inflammation index and systemic immune-response index in the prediction of adverse outcomes in pregnant women with antiphospholipid syndrome. Am. J. Reprod. Immunol..

[13] von Elm E., Altman D.G., Egger M., Pocock S.J., Gøtzsche P.C., Vandenbroucke J.P., STROBE Initiative (2007). The Strengthening the Reporting of Observational Studies in Epidemiology (STROBE) statement: Guidelines for reporting observational studies. Lancet.

[14] Collins G.S., Moons K.G.M., Dhiman P., Riley R.D., Beam A.L., Van Calster B., Ghassemi M., Liu X., Reitsma J.B., van Smeden M. (2024). TRIPOD+AI statement: Updated guidance for reporting clinical prediction models that use regression or machine learning methods. BMJ.

[15] Collins G.S., Dhiman P., Ma J., Schlussel M.M., Archer L., Van Calster B., Harrell F.E., Martin G.P., Moons K.G.M., van Smeden M. (2024). Evaluation of clinical prediction models (part 1): From development to external validation. BMJ.

[16] Zhu J., Li Z., Deng Y., Lan L., Yang J. (2024). Comprehensive reference intervals for white blood cell counts during pregnancy. BMC Pregnancy Childbirth.

[17] Puissegur A., Accoceberry M., Rouzaire M., Pereira B., Herault M., Bruhat C., Delabaere A., Gallot D. (2023). Risk factors for perineal wound breakdown in early postpartum: A retrospective case–control study. J. Clin. Med..

[18] Marchi F., Pylypiv N., Parlanti A., Storti S., Gaggini M., Paradossi U., Berti S., Vassalle C. (2024). Systemic immune-inflammation index and systemic inflammatory response index as predictors of mortality in ST-elevation myocardial infarction. J. Clin. Med..

